# Detecting the effects of opencast mining on ecosystem services value in arid and semi-arid areas based on time-series remote sensing images and Google Earth Engine (GEE)

**DOI:** 10.1186/s12862-024-02213-6

**Published:** 2024-02-29

**Authors:** Qinyu Wu, Liya Yang, Jiaxin Mi

**Affiliations:** 1 School of Public Policy & Management, China University of Mining and Technology, Xuzhou, 221116 China; 2 Key Laboratory of Mine Ecological Effects and Systematic Restoration, Ministry of Natural Resources, Beijing, 100081 China

**Keywords:** Ecosystem services value, Mining impact, Google earth engine platform, Sen + MK test, Joinpoint regression model, Ordos opencast mining areas

## Abstract

Ecosystem Services Value (ESV) are the various beneficial functions and products that natural ecosystems provide to humans, and are important indicators for evaluating ecosystem conditions and human well-being. Opencast mining is one of the human activities that severely damage the surface environment, but its long-term impact on ecosystem services lacks systematic assessment. This study takes the Ordos opencast mining area as an example, and calculates the value of ESV from 1990 to 2020 based on the Google Earth Engine platform. Mann-Kendall Tau-b with Sen’s Method (Sen + mk test) and Joinpoint regression model were used to analyzes its spatiotemporal variation characteristics. Further revealed the impacts of opencast mining on ESV as well as the trend of ESV changes. The results show that: (1) The dynamic ESV levels in the study area fluctuated considerably from 1990 to 2020 with an overall decreasing trend of 89.45%. (2) Among nine types ecosystem services, most of them were significantly different (*p* < 0.001) between mining areas and control areas, with biodiversity protection (BP), climate regulation (CR), gas regulation (GR), soil formation and retention (SFR), water supply (WS) and waste treatment (WT) showed a significant decrease between 1990 and 2020. (3) In the past 30 years, the ESV of the study area showed an overall improvement trend, where the improved area accounted for 48.45% of the total area of the study area. However, the degraded area also accounted for 21.28, and 17.19% of the area belonged to severe degradation. With 67% of the significantly degraded areas distributed within mining concessions. (4) The trend of ESV changes in the mining impact areas and the control area showed significant differences. The ESV of the control area increased continuously, with an average annual percentage change (AAPC) of 0.7(95%CI:0.50 ~ 0.9, *P* < 0.001) from 1990 to 2020; while the ESV of the mining impact areas first stabilized and then decreased significantly, with an AAPC of − 0.2(95%CI:− 0.3 ~ − 0.1,*P* < 0.001) from 1990 to 2020. This study provides scientific support for formulating ecosystem management, restoration plans, and payment for ecosystem service policies, which is conducive to achieving regional sustainable development and improving human well-being.

## Background

Opencast mining, especially surface mining, is one of the most intense human disturbances to terrestrial ecosystems, often causing drastic changes in Ecosystem Services [1]. Opencast mining destroys the surface soil, vegetation, topography and other features, results in removal or modification of areas of natural ecosystems [2], affecting their capacity to supply Ecosystem Services Value (ESV). In China, the areas of opencast mining reached 4746 km^2^ in 2015, with an increase by 2.7 times after 2000, most of which are concentrated in arid and semi-arid regions, where ESV have declined to varying degrees in the mining areas [3]. Understanding the effect of opencast mining on ESV can provide guidance for ecological restoration in mining areas, and also provide a basis for sustainable management of mining areas.

Opencast mining throughout the life of mine, from exploration and development to post-closure, have been impacting the ESV in mining areas. In the early stage of mining, the construction of infrastructure and roads will cause changes in land use, especially affecting the provisioning and regulating services of ecosystems [4]. During the mining process, on the one hand, mining causes direct damage to the surface ecosystem [3], on the other hand, it also disrupts the original water cycle process, leading to problems such as reduction of surface water system [5], decline of groundwater level [6], etc., resulting in the decline of ecosystem regulating and supporting services. After the mining is over, some residual waste rock piles, residues, etc. may produce water pollution and air pollution problems [7] throughand leaching processes, and may also cause spontaneous combustion of coal gangue [8], further affecting the ESV in mining areas. On the other hand, land reclamation, ecological restoration, natural vegetation recovery and other processes will also lead to the recovery of ESV in mining areas [9]. Therefore, the impact of opencast mining on ESV in mining areas is a long-term process, showing differentiated trends due to the mining life cycle.

The assessment of ESV in mining areas is the premise of exploring the impact of opencast mining. ESV assessment is a hot topic in recent decades, and models such as InVEST [10], ARIES [11], MIMES [12] model have been developed. Many scholars have applied these models to assess the impact of mining on ESV in mining areas. Four ecosystem services of Curragh mine including carbon sequestration, air quality regulation, soil conservation and water yield were assessed in 1989, 1997, 2005 and 2013, and changes in ESV caused by mining disturbances were mapped with LandTrendr algorithm [13]. In Shengli coalfield, Inner Mongolia, remote sensing images from 2000, 2005, 2010, 2015, and 2020 were used to evaluate the ecological service value based on land use, and surface coal mining impacts on ESV were analyzed [14]. Six typical opencast mining areas in the Southern Slope of Qilian Mountain were selected and the ESV of mining areas from the 1975 to 2016 were assessed, showing that the regional ESV decreased with the expansion of mining areas [1]. However, most of the previous studies reflected the impact of opencast mining on ESV in mining areas by changes in ecosystems in individual years, while neglecting that ESV in mining areas are a dynamic process, and few studies revealed the differences in the impact of different mining periods on ESV, making it difficult to reveal the regularity of opencast mining on ESV.

mineral resources are crucial for the survival and development of society, providing over 95% of the world’s energy, more than 80% of the world’s industrial raw materials, and over 70% of the world’s agricultural raw materials [15]. Most of the open-pit coal mines are located in arid and semi-arid ecologically fragile areas [9]. Opencast mining further deteriorates the ecological environment of the area, causing serious negative impacts on the economic, social development and well-being of the residents. This study selected two typical mining areas in China’s arid and semi-arid regions - Dongsheng mining area and Junggar mining area as the research objects, which are facing the dual pressure of ecological fragility and high-intensity resource exploitation. However, previous studies have paid insufficient attention to this field. The purpose of this study is: (1) Based on the GEE platform and long-term remote sensing data, to quantify the value, total static value and total dynamic value of nine ecosystem services in the study area from 1990 to 2020, We delineated mining impact and control areas according to administrative boundaries and mining concessions and compared the differences between them with statistical analyses, to understand the impact of opencast mining on the ESV; (2) To evaluate the dynamics of ecosystem services by using Mann-Kendall Tau-b with Sen’s Method (Sen + mk test) [16], and to analyze the value trends by using Joinpoint modeling, to compare the differences between the mining areas and the control areas, to explore the impact of opencast mining on the trends of ecosystem services. This paper is expected to reveal the dynamic changes and temporal differences of the value of ecosystem services in the opencast mining areas, and to provide theoretical basis for the ecological protection and restoration of the mining areas.

## Method

### Study area

**Fig. 1:**
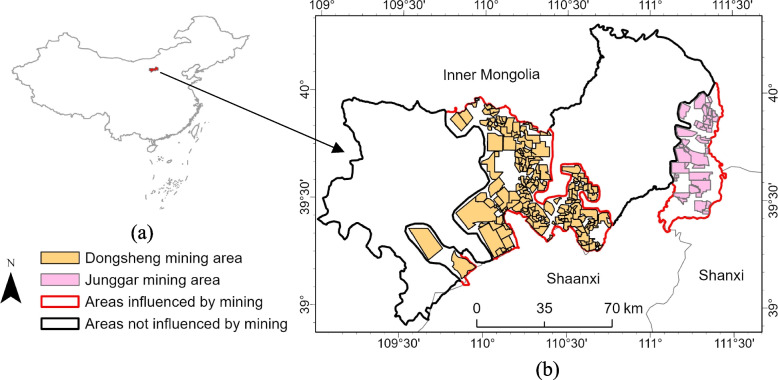
The location of the study area: **a** vicinity map showing the location of the study area in Junggar Banner, Dongsheng District and Yijinhuoluo Banner of Ordos City, Inner Mongolia, China, within an outline map of the provinces and administrative regions of China; **b** location of the mining concessions within the study area

The study area is located in Dongsheng District, Ordos City, Inner Mongolia Autonomous Region, China, in Junger and Yijinholo Banners, where the Dongsheng and Junger coal fields are distributed, and the mining method is mainly opencast mining (Fig. 1). From 2002 to 2012, rising coal prices in China drove the large-scale development of coal mines, and as of 2023, most of the mines are still under development, but the mining areas are also undergoing gradual ecological restoration. In order to compare different regions, the impact boundaries of the mining areas and the non-mining areas were divided according to the administrative boundaries and the mining concessions. Assuming that the mining and non-mining areas are comparable in other aspects except for mining activities, the non-mining area serves as valid control areas to assess the environmental impact of mining activities. The development of mining concessions can lead to drastic changes in land cover, destroying the original habitat. The region is located in the northwest of China, belonging to an arid and semi-arid climate, with low annual precipitation, vegetation cover mainly consisting of shrubs and sparse forest land, and coupled with the impact of coal mining, the ecological environment is fragile [17–19].

### Framework

**Fig. 2:**
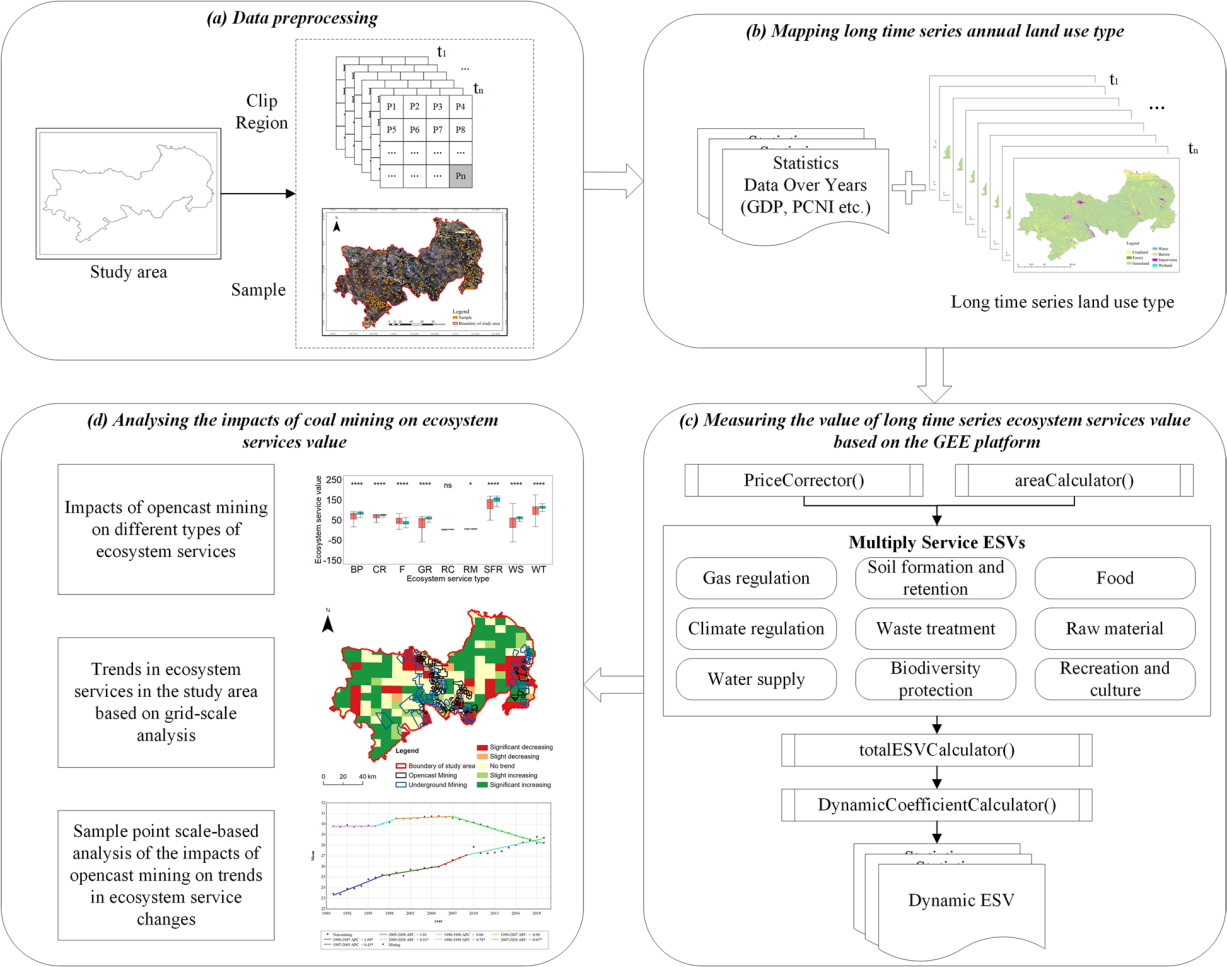
The framework of this study: (**a**) data processing; (**b**) mapping long time series annual land use type; (**c**) measuring the value of long time series ESV based on the GEE platform; (**d**) analysing the impacts of opencast mining on ESV

In this study, based on the Google Earth Engine platform and remote sensing data, the nine ecosystem services value, static totals and dynamic totals were calculated for the study area from 1990 to 2020, and the calculations were performed at both the grid scale and the sample point scale. The study then compares the differences in the nine ecosystem service values and dynamic totals and their trends between mining impact areas and control areas, the study framework diagram is shown in Fig. 2.

### Data resources

The land use classification data for this study came from the CLCD dataset developed by Yang Jie and others from Wuhan University [20]. When using this dataset in this study, the types were reclassified according to the actual situation of the study area, which was obtained through Google Earth high-definition imagery. Shrub types were removed from the data and frozen tundra and bare leakage land were combined as unused land based on the actual conditions in the study area, the reclassification of the data was realized by GEE. Our statistics of other parameters for ESV, like GDP, yearly GDP index, and PCNI, were extracted from the Inner Mongolia Ordos Bureau of Statistics,1990–2020. The data used in this study are shown in Table 1.

**Table 1:** Variables used to calculate the value of ecosystem services

Data application	Unit	Source
Delineate Land Systems	Class Index (0–6)	Yang and Huang. (2021)
Gross Domestic Product (GDP)	CNY/km^2^	Inner Mongolia Ordos Bureau of Statistics,1990–2020 (https://tjj.ordos.gov.cn/)
Per Capita Net Income(PCNI)	CNY	

### Ecosystem services value estimation

#### Assignment of ESV

Based on the studies of Costanza, d’Arge et al. [21] and Xie et al. [22], we adapted the ecosystem service equivalent weight coefficients in our application to the unique ecological and mining development conditions of the study area. Among the eight LULC types in our map, shrub type represents the land covered by shrubs, short trees and orchards growing on the plain, and its ecological function is similar to that of shrubs. Impervious surfaces have a negative impact on three ecological functions, such as water supply, waste treatment and other ecosystem services, which reduce the overall ESV of the entire ecosystem [23].

Table 2 lists the weights of the land use types mentioned in this paper for ecosystem services. According to the data provided by the Inner Mongolia Statistical Yearbook, the average actual grain yield of cultivated land from 1990 to 2020 was 4405 kg/ha, and the average grain price in 1990 was 3.05 CNY/kg. Therefore, the ESV per unit area (per hectare) was 1919.32 CNY. Subsequently, Table 3 shows the unit area value coefficients of each LULC in ordos.

**Table 2:** Equivalent weight factor of ecosystem services per hectare in Ordos [22]

	Gas regulation	Climate regulation	Water supply	Soil formation and retention	Waste treatment	Biodiversity protection	Food	Raw material	Recreation and culture	total
Woodland	2.35	7.03	0.37	3.08	1.99	2.6	0.1	0.71	1.14	19.37
Grassland	0.51	1.34	0.08	1.95	0.44	0.56	0.3	0.14	0.25	5.49
Cropland	0.67	0.36	0.02	1.15	0.1	0.13	0.85	0.4	0.06	3.74
Wetland	1.9	17.1	15.5	2.49	3.6	7.87	0.51	0.5	4.73	54.2
Waterbody	0.77	14.6	20.4	1	5.55	2.55	0.8	0.23	4.34	50.24
Unused land	0	0	0.02	0.14	0.01	0.12	0.01	0.03	0.01	0.34
Build up	−2.42	0	−7.51	0.02	−2.46	0	0	0	0	−12.37

**Table 3:** ESV of per hecta re (unit area) of different LULC categories in Ordos (CNY/ha)

	GR	CR	WS	SFR	WT	BP	FP	RM	RC
Woodland	4510.402	13,492.8196	710.1484	5911.5056	3819.4468	4990.232	191.932	1362.7172	2188.0248
Grassland	978.8532	2571.8888	153.5456	3742.674	844.5008	1074.8192	575.796	268.7048	479.83
Orchard	1285.9444	690.9552	38.3864	2207.218	191.932	249.5116	1631.422	767.728	115.1592
Cropland	3646.708	32,820.372	29,749.46	4779.1068	6909.552	15,105.0484	978.8532	959.66	9078.3836
Wetland	1477.8764	28,022.072	39,154.128	1919.32	10,652.226	4894.266	1535.456	441.4436	8329.8488
Waterbody	0	0	38.3864	268.7048	19.1932	230.3184	19.1932	57.5796	19.1932
Unused land	− 4644.7544	0	−14,414.0932	38.3864	− 4721.5272	0	0	0	0
Build up	4510.402	13,492.8196	710.1484	5911.5056	3819.4468	4990.232	191.932	1362.7172	2188.0248

#### Calculation of ESV

Once the average annual ecosystem service value per unit area of each LULC category has been determined, the ESV for the study area can be calculated using the following formula:

1 $${ESV}_f={\sum}_f{A}_k\times {VC}_{kf}$$

2 $${ESV}_k={\sum}_k{A}_k\times {VC}_{kf}$$

3 $${ESV}_s={\sum}_k{\sum}_f{A}_k\times {VC}_{kf}$$

*ESV*_*K*_, *ESV*_*f*_, *ESV*_*s*_ refer to the ESVs of LULC category *k*, service type *f*, and the total static ecosystem value. *A*_*k*_ represents the areas of LULC category *k*, and *VC*_*kf*_ is the value coefficient (CNY/ha/a) for category k and service function type *f*.

#### Dynamic adjustment of ESV

To ensure the comparability of all ESVs at different times, it is necessary to calculate the equivalent economic value, which accounts for the effects of price level, inflation and other factors. In this study, Ordos depends on coal mining resource development as a pillar industry, which exerts a huge impact on GDP. we use the following equation to calculate the equivalent economic value, and we set 1990 as the constant and base year for calculating the annual average ESV:

4 $${ESV}_C=\frac{ESV_S}{E_{avg}}\times {E}_{an}$$

5 $${E}_{an}={E}_m/\prod_{i=n}^m{\varnothing}_i$$

*ESV*_*c*_ is the comparable *ESV*. *E*_*avg*_, *E*_*an*_, *E*_*m*_ are economic values of one weight factor, while *E*_*avg*_ is the average evalue and *E*_*an*_ is calculated by the *E*_*m*_ in current year m during the study period, n refers to the constant year (start year here). ∅ is the yearly GDP index.

As the society develops and the living standard improves, people will pay more attention to ecosystem services, leading to their increased willingness to pay for ecosystem services. Therefore, the static ESV values should be adjusted by the socio-economic coefficient. Pearl’s S-shaped growth curve can be used to describe the characteristics of ecological value [24].

6 $${ESV}_d={ESV}_c\times {A}_c$$

7 $${A}_c=\frac{1}{1+{\mathit{\exp}}^{-t}}$$

8 $$t=\frac{1}{E_n}-3$$

*ESVd* refers to the dynamic *ESV*. *Ac* is the dynamic adjustment coefficient. *t* represents the social development stage. *En* is the Engel coefficient.

We modified the GEE code of Liang et al. [25] to calculate the value of the f-th ecosystem service, the ESVs and the ESVd of the study area using the API and the code editor on the Google Earth Engine platfrom. The calculation model included the following steps: (1) We customized the weight values of the equivalence coefficients; (2) We calculated the value of the type f ecosystem service and the total static ecosystem service value; (3) We calculated the total value of the dynamic ecosystem service value by integrating social development variables. In addition, ESV was calculated on a 10 km × 10 km grid scale. To compare ESV at mining impact areas with those at control areas, 300 sample points were randomly distributed within the study area (see Fig. 4(a)). A 1 km buffer zone was created around each sample point, and the value per unit area within the buffer zone was calculated as the value for the sample point.

### Mann-Kendall tau-b with Sen’s method

Sen + MK test can detect the strength of the change in ESV. Sen slope estimator is used to calculate the trend value of ESV change, which is usually combined with MK nonparametric test, that is, first calculate the Sen trend value, and then use MK method to judge the trend significance [5, 26].

Theil-Sen Median trend analysis (Sen trend analysis) is a robust nonparametric statistical method for trend calculation. Compared with linear regression trend analysis, Sen trend analysis can avoid the influence of missing data and data distribution shape in time series, and eliminate the interference of outliers on time series. The calculation formula of Sen trend degree is shown in formula (1).

9 $${\beta}_{\textrm{ESV}}=\textrm{median}\left(\frac{ESV_{dj}-{ESV}_{di}}{j-i}\right),\forall j>i$$

In the formula, *ESV*_*dj*_ and *ESV*_*di*_ are the time series data of *ESV*_*d*_. When *β*_ESV_ > 0, it means that ESV shows an upward trend; when *β*_ESV_ < 0, it means that ESV shows a downward trend.

Mann-Kendall test (MK test) is usually used in combination with Sen trend analysis. This method is a nonparametric statistical test method, which is not affected by missing values and outliers, and does not require the sample data to follow a certain distribution. The statistical test method is shown in formulas (2) to (5).

10 $$Z=\left\{\begin{array}{cc}\frac{S-1}{\sqrt{Var(S)}}& \left(S>0\right)\\ {}0& \left(S=0\right)\\ {}\frac{S-1}{\sqrt{Var(S)}}& \left(S<0\right)\end{array}\right.$$

11 $$\textrm{S}=\sum_{j=1}^{n-1}\sum_{i=j+1}^n\mathit{\operatorname{sign}}\left({ESV}_{dj}-{ESV}_{di}\right)$$

12 $$\textrm{Var}\left(\textrm{S}\right)=\frac{n\left(n-1\right)\left(2n+5\right)}{18}$$

13 $$\operatorname{sign}\left(\uptheta \right)=\left\{\begin{array}{cc}1& \left(\theta >0\right)\\ {}0& \left(\theta =0\right)\\ {}-1& \left(\theta <0\right)\end{array}\right.$$

In the formula, ESV_*dj*_ and ESV_*di*_ are the time series data of *ESV*_*d*_; *sign* is the sign function; *S* is the test statistic; *Z* is the standardized test statistic, Used to test whether there is a significant trend in the time series data, the larger the Z-value, the more significant the trend [27, 28]. n is the number of data. Given the significance level α, if |*Z*| > *Z*_1 − *α*/2_, it indicates that there is a significant trend change. In this study, *α* = “0.05”, that is, to judge the significance of the trend change of ESV time series at the 0.05 significance level.

### Joinpoint regression model

Joinpoint regression, also known as segmented regression, piecewise regression, etc., is essentially to establish segmented regression based on the time characteristics of sample distribution, divide the research time into different intervals by several connecting points, and perform curve fitting and optimization for each interval, and then evaluate the variable change characteristics of different intervals with specificity within the global event range in more detail. There are two types of joinpoint regression models: linear model (*y = xb*) and log-linear model (*ln y = xb*). In this study, the log-linear model is selected, and the regression equation is as follows:

14 $$\mathit{\ln}{y}_t={\beta}_0+{\beta}_1t+\sum_{i=1}^{k-1}{\beta}_{i+1}{\left(t-{T}_i\right)}_{+}+{\varepsilon}_t$$

where *y*_*t*_ is the dependent variable, *t* is the independent variable (time), k is the number of breaks, *T*_*i*_ is the time point of the *i*-th break, (*t* − *T*_*i*_)_+_ is the positive part of (*t* − *T*_*i*_), *β*_0_ is the intercept, *β*_1_ is the slope of the first segment, *β*_*i* + 1_ is the change in slope at the *i*-th break, and *ε*_*t*_ is the error term.

The join points play a key role in connecting different segments in the join point regression model. In this study, we used Joinpoint5.0.2 software, which is free software developed and maintained by the National Cancer Institute of the United States, to perform the joinpoint regression analysis. This software can automatically fit and compare models with different numbers of join points and provide rich graphical and tabular outputs. The software can be downloaded from the website: Joinpoint Regression Program (cancer.gov). We use Weighted BIC method to select the model, that is, the number of joinpoints.

Annual percentage change (APC) is a method to represent the trend of ESV change over time, that is, the joinpoint regression model fits the connected log-linear segments, and the calculated APC value is used to indicate the rate of constant percentage change of ESV in each segment on a logarithmic scale. Average annual percentage change (AAPC) is a summary measure of the change trend within a fixed time segment specified in advance by the model, that is, using specific numbers to describe the average APC of all observation times. The last step of joinpoint regression model calculation is to convert the weighted average of slope linear coefficients into annual percentage change. In nonlinear models, the calculation formulas of APC and AAPC are as follows

15 $$\ln \left(y|x\right)={b}_o+{b}_{1x}$$

16 $$APC=\left\{\exp \left({b}_1\right)-1\right\}\times 100\%$$

17 $$AAPC=\left\{\exp \left(\frac{w_i{b}_i}{w_i}\right)-1\right\}\times 100\%$$

In the formula, *x* is the year, *y* represents *ESVd*, *b*_*0*_ is the outcome, *b*_*i*_ is the regression coefficient, that is, the slope coefficient of each segment, *w*_*i*_ is the number of years contained in each time segment.

In this paper, if the calculation result shows that APC value is greater than 0, it means that the ESVd of the study area shows a downward trend during that time segment. If APC = AAPC, it means that the number of joinpoints selected by the optimal model of the software is 0. In this case, the 95% confidence interval (95%CI) of APC value can be used to judge whether the trend change in that period segment has statistical significance. The calculation involves all test levels of α = 0.05.

### Statistical analysis

The sample points of the mining impact areas and the control areas were 268 and 242 respectively. In this study, nonparametric test (Mann-Whitney U) was used to identify whether there was a significant difference between the mining impact areas and the control areas at the confidence level of 0.05. All data were analyzed by SPSS and Matlab software; in addition, this study used R language to extract the average level, fluctuation degree and outliers of 9 kinds of ecosystem services GR, CR, WS, SFR, WT, BP, FD, RM, RC of all sample points in 1990, 2000, 2010 and 2020.

## Results

### Spatiotemporal changes of ESV in opencast mining areas

**Fig. 3:**
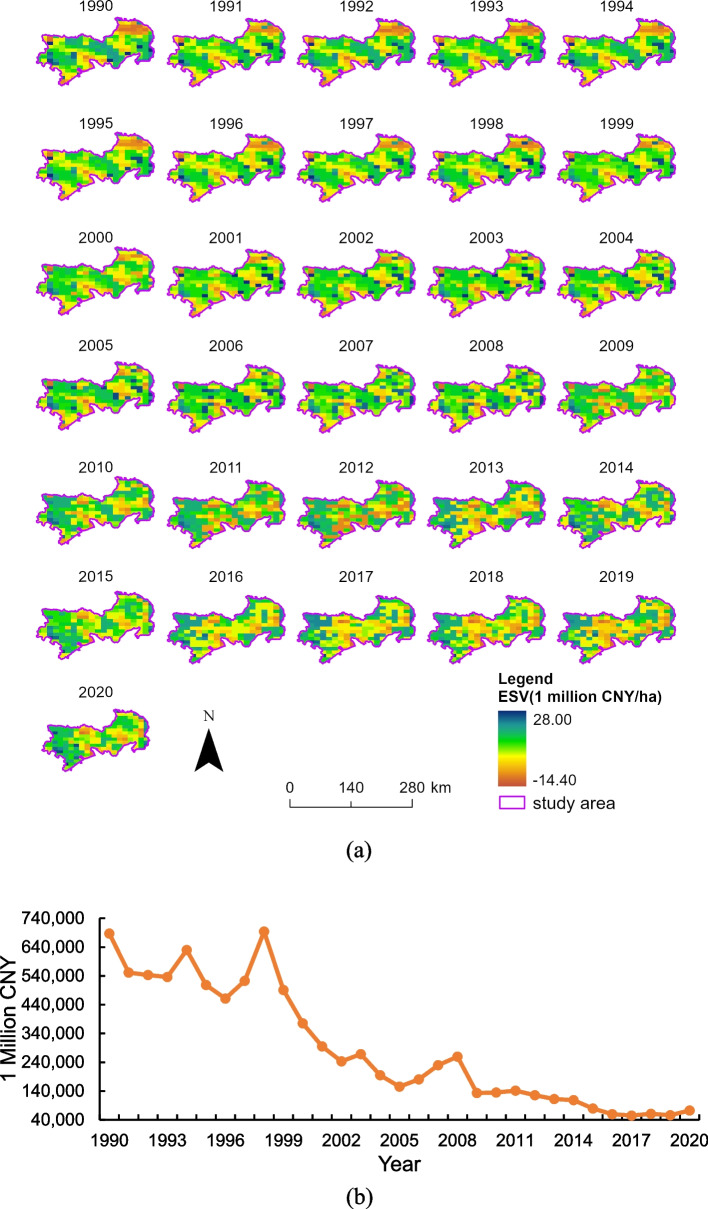
ESV in the study area, 1990–2020. **a** Temporal and spatial distribution pattern of ESVd per hectare (**b**) Changes in total ESVd values in the study area

Figure 3(a) illustrates the spatial pattern of dynamic ESVd per hectare from 1990 to 2020. The spatial pattern of dynamic ESV is consistent and locally variable. The high-value zones are primarily located in the northwestern and southeastern parts of the study area, while the low-value zones are mainly situated in the northeastern, central, and southern parts of the study area. The low-value zones in the northeastern part of the study area changed gradually from strips to patches. In contrast, the low-value zones in the central part of the study area showed a gradual shift towards contiguous patches. Figure 3(b) shows that the total dynamic ESV levels in the study area decreased significantly, first decreasing, then increasing, and then decreasing again, with a general trend of decline. The average value decreased from 687,245.13 CNY in 1990 to 72,442.34 CNY in 2020, representing an 89.45% decrease.

### The impact of opencast mining on different types of ESV

**Fig. 4:**
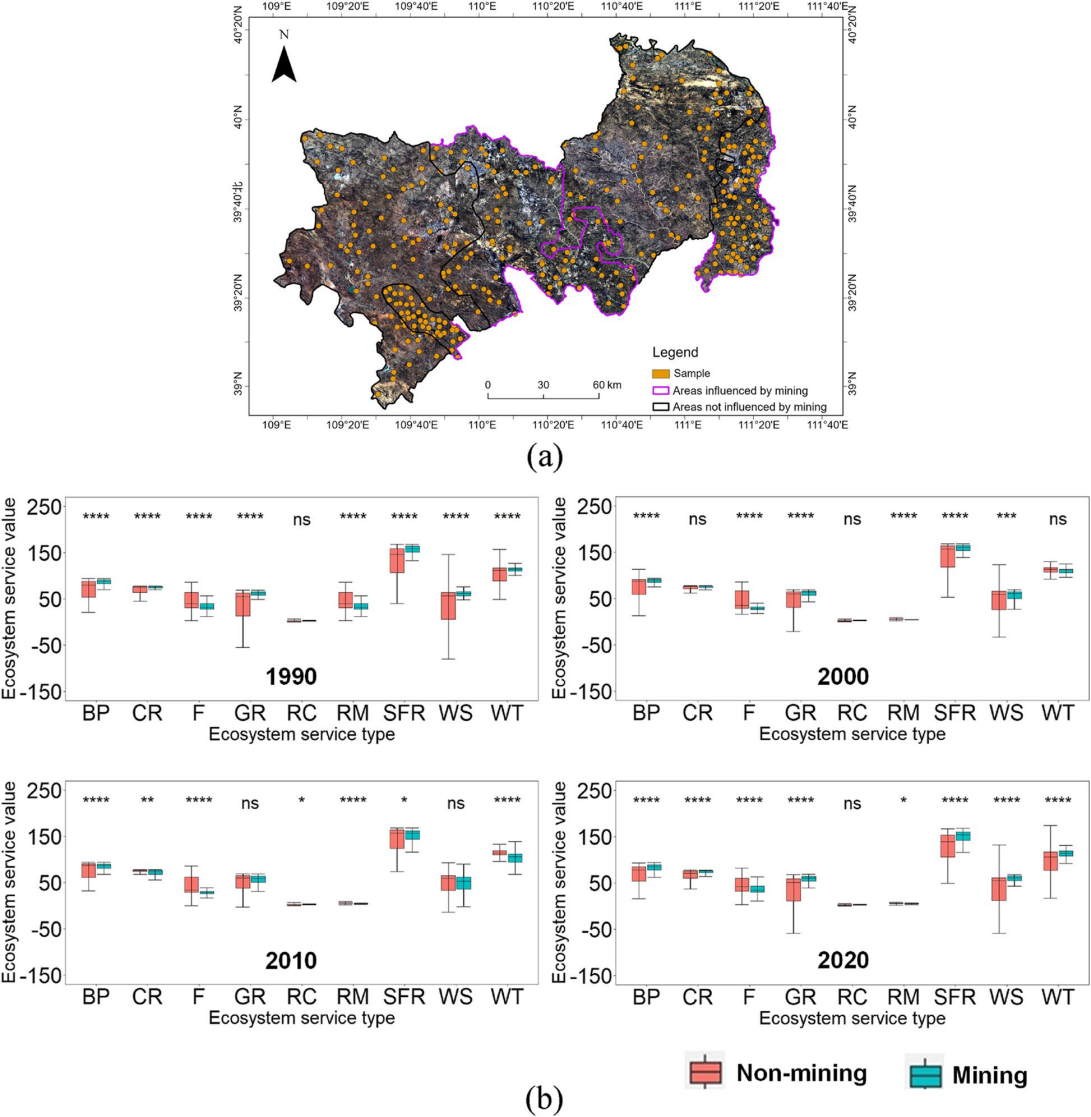
**a** Sample points; **b** Boxplots of nine types ecosystem service value in mining impact areas and control areas

The aim of this section is to explore the differences in ecosystem service value for each type in mining impact and control areas. As shown in Fig. 4(b), RC had the lowest median values in 1990, 2.67 and 2.47 for impact and control areas, respectively, which increased to 3.62 and 5.15 by 2020. RM had median values that were only slightly higher than those of RC, about 4.81 and 5.12 in 1990, but decreased to 4.78 and 3.78 by 2020. The other ecosystem service types (BP, CR, F, GR, SFR, WS and WT) had higher values, with SFR having the largest share in the graph.

For the type of ESV, in 1990, the median ESV in mining impact areas was slightly higher than that in control areas, except for F and RM. The median values of SFR were 153.94 and 139.07 for mining impact and control areas, respectively. However, the range of fluctuations in values for control areas was much greater, with quartile distances ranging from 49.54 (WS) to 1.09 (RM), while the interquartile range for the mining impact areas was only between 18.00 (SFR) and 0.48 (RM). An independent t-test was performed to test whether there was a significant difference between the mining impact and control areas. The results showed that there was a significant difference, as the *p*-values for BP, CR, F, GR, RC, RM, SFR, WS and WT were all less than the 0.001 level of significance. However, by 2020, the mean values of BP, CR, GR, SFR, WS and WT in the control areas exceeded those in the mining impact areas. The median values of SFR were 156.72 and 157.24 for the mining impact and control areas, respectively. The median values of WS were 53.33 and 58.62, and those of WT were 106.03 and 113.07, respectively. The quartile spacing remained stable from 1990 to 2020. The results of the significance test showed that the *p*-values of BP, CR, F, RC, RM, SFR and WT were less than the 0.05 level of significance and indicated significant differences.

### The impact of opencast mining on the trend of ESV changes

#### The trend of ESV changes in the opencast mining areas

**Fig. 5:**
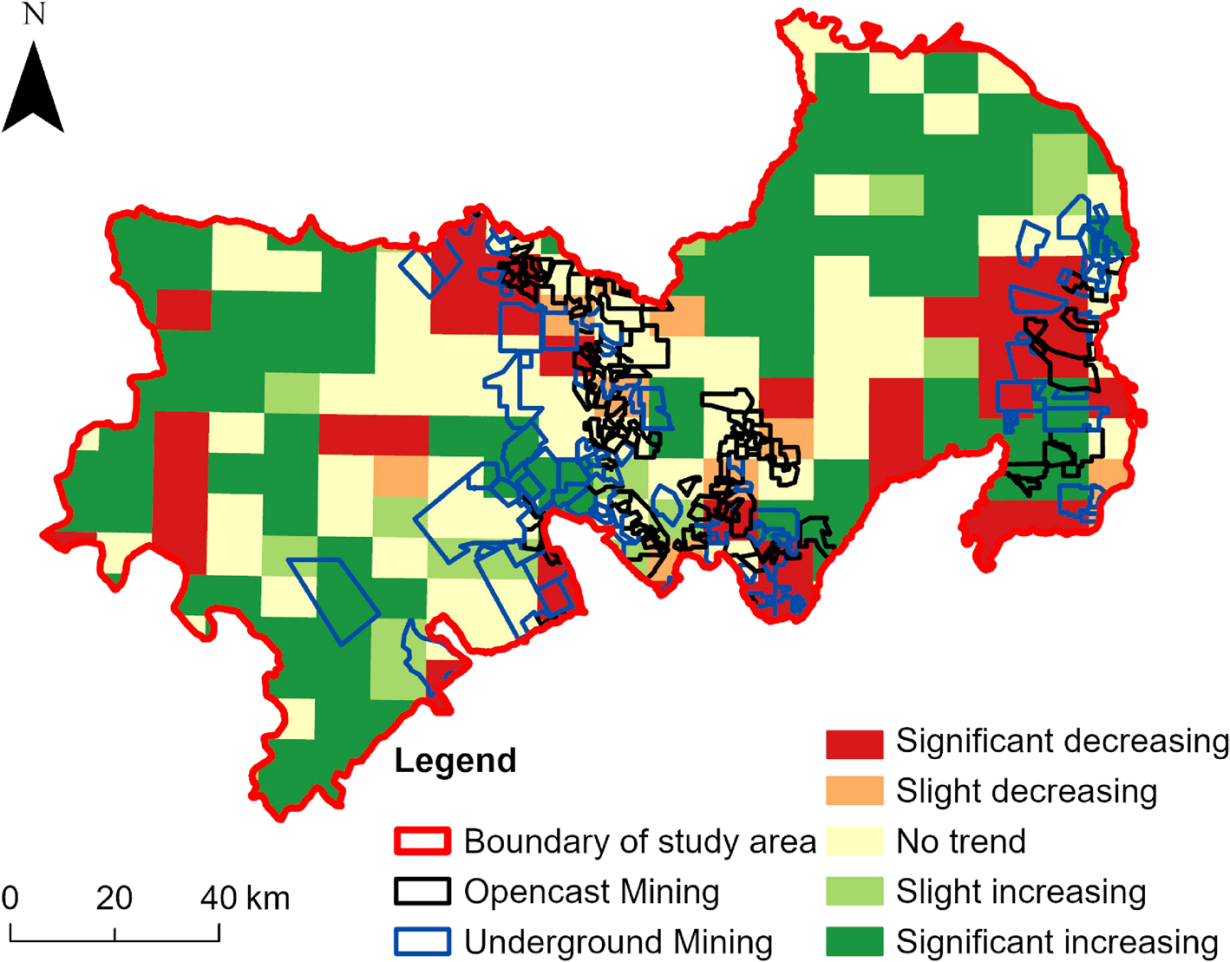
The trend of ESV in the study area, 1990–2020

We used the Sen + MK test to calculate the spatial distribution of the trend of ESV changes based on the grid scale, as shown in Fig. 5, during the period 1990–2020, the ESV in the study area showed an overall improving trend. The areas with improved ESV were mainly distributed in the western and north-eastern parts of the study area, while the areas with degraded ESV were mainly located in the south-eastern, north-central and south-central parts, which had a high degree of overlap with the distribution of mining concessions; statistical results show that 67% of the extremely degraded areas in the study area are located within the boundaries of mining concessions. Table 5 summarises the areas of each type of ESV change from 1990 to 2020. It can be seen that the areas of improvement were much larger than the areas of degradation during the study period. Among them, the areas of significant ESV degradation accounted for 17.19% of the total areas of the study area, and slight degradation accounted for 4.09%; the areas of slight ESV improvement accounted for 7.30%, and significant improvement accounted for 41.15%; and the areas of stable and unchanged ESV accounted for 30.27%.

**Table 5:** The areas and proportion of ESV change trends from 1990 to 2020

*β*_*ESV*_	Z	Type of change trend	Areas/km^2^	Percentage/%
*β*_*ESV*_ < − 0.005	|*Z*| ≥ 2.58	Significant increasing	6397.96	41.15%
*β*_*ESV*_ < − 0.005	|*Z*| < 2.58	Slight increasing	1134.87	7.30%
0.005 ≤ *β*_*ESV*_ < 0.005		No trend	4706.67	30.27%
*β*_*ESV*_ > 0.005	|*Z*| < 2.58	Slight decreasing	636.30	4.09%
*β*_*ESV*_ ≥ 0.005	|*Z*| ≥ 2.58	Significant decreasing	2672.93	17.19%

#### The trend of ESV changes in the coal mining impact areas

**Fig. 6:**
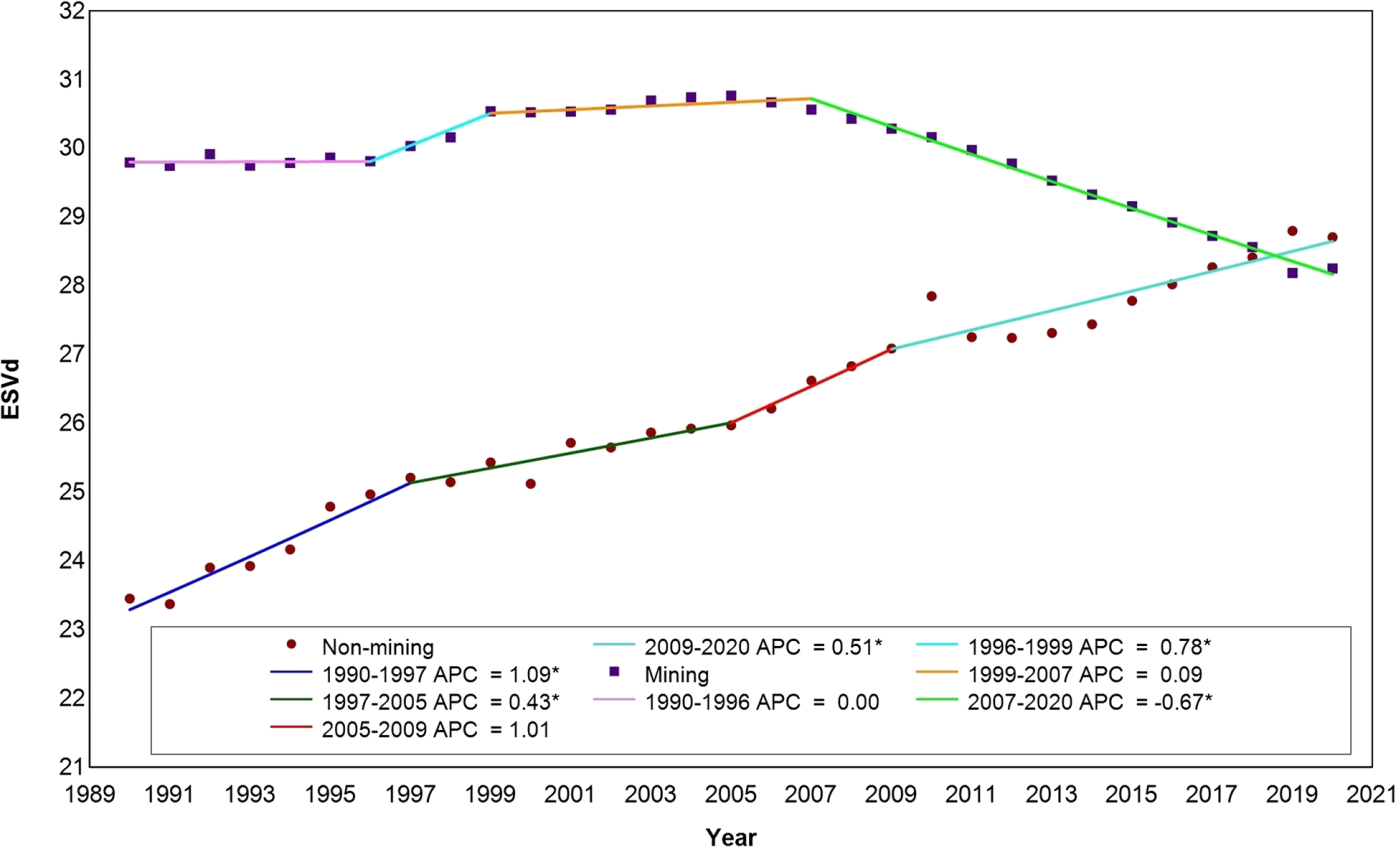
Trend results of Joinpoint regression analysis of ESVd in mining impact areas (Mining) and control areas (Non-mining), 1990–2020. APC, annual percentage change; *, statistically significant, *p* < 0.05

As shown in Fig. 6, for the control areas, there were three turning points in 1997, 2005 and 2009, the APC (95% CI) corresponding to the four intervals were APC1990–1997 = 1.09 (95% CI:0.7 ~ 1.5, *P* < 0. 001), APC1997–2005 = 0.43 (95% CI:0.8 ~ 2.4, *P* = 0.028), APC2005–2009 = 1.01 (95% CI:-0.3 ~ 2.3, *P* = 0.122), APC2009–2020 = 0.51 (95%CI:0.3 ~ 0.7, *P* < 0.001), and global AAPC1990–2020 = 0.7 (95%CI:0.5 ~ 0.9, *P* < 0.001). The global AAPC results showed that ESV in control areas in Erdos increased by an average of 0.7% per year during 1990–2020. Regarding the mining impact areas, the value of ecological system service showed three turning points in 1996, 1999 and 2007, corresponding to the four intervals of APC (95% CI) were APC1990–1996 = 0.00(95% CI:-0.1 ~ 0.1, *P* = 0.934), APC1996–1999 = 0.78(95% CI:0. 1 ~ 1.5, *P* = 0.03), APC1999–2007 = 0.09 (95%CI:0 ~ 0.2, *P* = 0.062), APC2007–2020 = − 0.67 (95%CI:-0.7 ~ − 0.6, *P* < 0.001) and global AAPC1990–2020 = − 0.2 (95%CI:-0.3 ~ − 0.1, *P* < 0.001), indicating that the ESV in the mining impact areas decreased by an average of 0.2% per year. Overall, the trends were markedly different between the mining impact areas and the control areas, and the year 2008 marked the point at which the control areas overtook the mining impact areas.

## Discussion

### The impact of coal mining on ESV

With the rapid development of China’s economy, China’s mining industry (especially coal) has grown dramatically, driving the large-scale changes in land use and land cover (LULC) in the Ordos mining areas. In the [17] past 30 years, natural ecosystems, especially grasslands that are common in the agro-pastoral transition zone in northern China, have been fragmented and degraded due to the rise of mining and related land use [29]. The economic development brought by mining has generated spillover effects, increasing the built-up areas, especially near the mining areas, which is a characteristic of LULC change types in resource areas. Compared with grasslands, the decline trend of forest cover in the study area was not obvious, which mainly benefited from the forest protection policies of the national and local governments [30]. At the same time, the gradual restoration of forestland increased the vegetation cover range.

From 1990 to 2020, the trend of ESVd (Ecosystem Service Value decline) in the study area exhibited significant spatial heterogeneity. The regions experiencing substantial decreases in ESVd were closely associated with mining development areas. In our analysis, we treated mining impact areas and control areas separately. However, it’s essential to recognize that this approach assumes uniform distribution of mining impact due to the extensive range and degree of influence from opencast coal mining on the surrounding ecological environment. One limitation of this assumption lies in its neglect of spatio-temporal correlation within the impact areas. The ESVd values for each assessment unit are not independent; they exhibit correlations across different temporal and spatial positions. Ignoring these spatial and temporal effects can introduce biased standard errors and lead to inaccurate significance tests [31, 32]. To gain a deeper understanding of the spatial dynamics, we propose employing a spatial dependency framework. For instance, the Spatial Error Model (SEM) accounts for spatial dependence in observational data errors [33]. By doing so, one can better reveal the spatio-temporal differences in impact, ultimately enhancing the quality and effectiveness of evaluation.

Figure 6 illustrates a significant decrease in mining concessions from 2010 to 2020, aligning with the rapid expansion of mining areas. Notably, 67% of the regions exhibiting significant declines (as shown in Fig. 5) fall within mining concessions, highlighting the negative impact of opencast mining on ecosystem services. While there has been a 41.15% improvement in ESV, driven by natural forests and incremental restoration efforts adopted by several mines, Fig. 3(b) reveals a significant overall decline in ESV across the entire region. This discrepancy suggests that ecological restoration efforts are still lagging behind the pace of mining development [3, 4, 34].

### The response of different ESV to opencast mining

Among the nine ESV in Ordos, SFR had the highest level, which might be because Ordos was mainly dominated by grasslands, accounting for more than 75% of the city’s area. Grasslands had a strong water conservation function, which could increase rainfall infiltration, reduce runoff and evaporation,, and promote groundwater recharge [35]. RC had the lowest level, which might be because there were no large-scale human landscape construction and tourism development projects in the city [36]. In addition, the raw material service in Ordos increased significantly from 1990 to 2000, which might be because the city implemented ecological construction measures such as returning farmland to forest, afforestation, etc., during this period, increasing the forestland area and forest stock volume [37].

From 1990 to 2020, coal mining had a certain impact on the nine ESV. Specifically, the ESV of the non-mining impact areas was higher than that of the mining impact areas during this period, indicating that the mining impact areas was under continuous ecological and environmental pressure during this period, such as land reclamation, water resource consumption, pollutant discharge, etc., which reduced the quality and function of the ecosystem. In addition, the fluctuation degree of data in the mining impact areas was obviously smaller than that in the control areas, which might be because the LULC types and ecosystem services functions in the non-mining impact areas were more diversified, and there were larger differences and trade-offs between different types of land and services. While the LULC types and ecosystem services functions in the mining impact areas were relatively simple, mainly dominated by grasslands, providing relatively single types of services.

### Implications for protection and restoration of arid and semi-arid mining ecosystems

In the early stage of coal mining, the ecosystem in the mining areas was mostly in the initial stage, and the original ecosystem was less affected by human activities. In the accelerated development and stable development stages of coal mining, ecological problems became prominent, and the ecosystem was seriously damaged. If ecological restoration was timely, it could achieve positive succession of the damaged ecosystem, or slow down the negative succession of the ecosystem [34]. However, if no measures were taken, under the current economic and technological conditions, the ecosystem would continue to degrade or regress to an extreme ecosystem. In the development decline and closure stages of coal mining, if ecological restoration was the main focus and economic development was the auxiliary, then the ecosystem in the mining areas would continue to recover [38, 39]. However, if relevant ecological restoration measures were reduced or cancelled until after the closure of the mine, then ecological problems in the mining areas would persist and spread, causing regional ecological problems after the closure of the mine. It can be seen that timely and reasonable human intervention and management can achieve positive succession of the ecosystem, that is, the restoration/reconstruction of the ecosystem [40]. This restoration and reconstruction can be a reset of the destroyed trajectory, or a new structure construction. The consequences can achieve complete or partial recovery of the structure and function of the original ecosystem, or establish a new stable ecosystem.

The reclamation and restoration of the mining areas should strengthen the restoration of Cr, Cd, Pb, Zn and Ni in the soil [35]. For reclaimed areas, adjust vegetation types appropriately and select economically suitable vegetation such as alfalfa, alkali grass, larch, caragana for planting and restoration; for unreclaimed spoil dumps, first carry out reclamation, then conduct heavy metal content detection and select suitable restoration vegetation [26]. In addition, in order to enhance the resilience of the ecosystem, it is also necessary to pay attention to the interaction between the ecosystem and human system [41]. It should be based on the comprehensive ability of various social subjects to cope with economic, ecological and other factors that damage society and fundamentally transform it. For example, government should conduct macro-regulation on mining areas from economic, social, ecological and other related policies; enterprises should seek green mining technology and carry out engineering transformation and construction of mining areas technical facility system with high productivity and low pollution; scientific research institutions should conduct continuous monitoring and evaluation on ecological security of mining areas; three parties should communicate with each other and form a “government-enterprise-scientific research” combined resilience regulation model.

## Conclusion

Evaluating the impact of opencast mining on ESV is of great significance for the management and sustainable development of mining impact areas. Taking Ordos coal mining areas as an example, this study measured the spatiotemporal changes of ESVd in the opencast mining areas areas for 30 years based on GEE platform, and analyzed the impact of coal mining on the *i-th* type ESV, as well as the impact on the trend of ESVd changes. This study can provide a method and reference for the long-term impact of ESV. The conclusions of this study include: (1) From 1990 to 2020, the dynamic ESV levels in the study area were fluctuating with an overall trend of decline. The mean value decreased from 687,245.13 Yuan in 1990 to 72,442.34 Yuan in 2020, with a decrease of 89.45%. (2) For the different subcategories of ecosystem services, most of them were significantly different (*p* < 0.001) between mining-impacted and non-impacted areas, with biodiversity protection (BP), climate regulation (CR), gas regulation (GR), soil formation and retention (SFR), water supply (WS) and waste treatment (WT) showed a significant decrease between 1990 and 2020. (3) In the past 30 years, the ESV of the study area showed an overall improvement trend, where the improved area accounted for 48.45% of the total area of the study area. However, the degraded area also accounted for 21.28, and 17.19% of the area belonged to severe degradation. Through the overlay analysis with the mining right boundary range, we found that most of the severely degraded areas are distributed within the mining concessions, Accounting for 67% of significantly degraded areas. (4) The trend of ESV changes in the mining-affected area and the non-affected area showed significant differences. The ESV of the non-affected area increased continuously, with an average annual percentage change (AAPC) of 0.7(95%CI:0.50 ~ 0.9, *P* < 0.001) from 1990 to 2020; while the ESV of the mining-affected area first stabilized and then decreased significantly, with an AAPC of − 0.2(95%CI:− 0.3 ~ − 0.1, *P* < 0.001) from 1990 to 2020. The turning point occurred in 2007, with an annual percentage change (APC) of − 0.67(95%CI:− 0.7 ~ − 0.6, *P* < 0.001) from 2007 to 2020.

The results of this study not only contribute to a clear understanding of the impact of opencast mining on the long-term trend of ESV changes, but also provide a basis for the formulation of spatially targeted ecosystem management, restoration plans and ES payment policies. This will help to maintain and improve human well-being while achieving regional sustainable development.

## Data Availability

The authors confirm that the data supporting the findings of this study are available within the article. Raw data that support the findings of this study area available from the corresponding author upon responsible request.

## Abbreviations

ESV: Ecosystem services value
ESVs: Static ecosystem services value
ESVd: Dynamic ecosystem services value
CR: Climate regulation
RM: Raw materials
FP: Food production
GR: Gas regulation
WS: Water supply
WT: Waste treatment
BP: Biodiversity protection
SFR: Soil formation and retention
RC: Recreation and culture
